# Metadata analysis indicates biased estimation of genetic parameters and gains using conventional pedigree information instead of genomic-based approaches in tree breeding

**DOI:** 10.1038/s41598-022-06681-y

**Published:** 2022-03-10

**Authors:** Jean Beaulieu, Patrick Lenz, Jean Bousquet

**Affiliations:** 1 Canada Research Chair in Forest Genomics, Institute of Systems and Integrative Biology and Centre for Forest Research, Université Laval, 1030 Avenue de la Médecine, Quebec, QC G1V 0A6 Canada; 2 Natural Resources Canada, Canadian Wood Fibre Centre, Quebec, QC, G1V 4C7 Canada

**Keywords:** Ecology, Genetics, Plant sciences

## Abstract

Forest tree improvement helps provide adapted planting stock to ensure growth productivity, fibre quality and carbon sequestration through reforestation and afforestation activities. However, there is increasing doubt that conventional pedigree provides the most accurate estimates for selection and prediction of performance of improved planting stock. When the additive genetic relationships among relatives is estimated using pedigree information, it is not possible to take account of Mendelian sampling due to the random segregation of parental alleles. The use of DNA markers distributed genome-wide (multi-locus genotypes) makes it possible to estimate the realized additive genomic relationships, which takes account of the Mendelian sampling and possible pedigree errors. We reviewed a series of papers on conifer and broadleaf tree species in which both pedigree-based and marker-based estimates of genetic parameters have been reported. Using metadata analyses, we show that for heritability and genetic gains, the estimates obtained using only the pedigree information are generally biased upward compared to those obtained using DNA markers distributed genome-wide, and that genotype-by-environment (*G*x*E*) interaction can be underestimated for low to moderate heritability traits. As high-throughput genotyping becomes economically affordable, we recommend expanding the use of genomic selection to obtain more accurate estimates of genetic parameters and gains.

## Introduction

Forest tree breeding traditionally aimed to increase volume production and improve both adaptive traits and fiber attributes of forest tree plantations. Ongoing environmental and market changes are currently shifting the selection focus towards seedstock production for enhanced carbon sequestration capacities and resistance to biotic and abiotic stressors. Those multiple breeding goals and the long-lived perennial nature of trees demand for the most precise estimation of genetic parameters and exact selection of individuals that best combine the desired properties of various nature (e.g.^1,2^). Compared with crop or animal breeding, forest tree breeders initiated their research activities quite recently, i.e., in the late 1950’s^3^. Since then, genetic improvement programs have been initiated for a large number of forest tree species from around the world^4^. The breeding process in forest tree species is often slow due to the limited resources available and to its inherent complexities. Among these complexities, are: (1) the necessity to set up more or less long-term progeny tests to assess the traits of interest, (2) the fact that many of these traits are often mature traits, thus requiring many years or even decades of testing before their appropriate assessment can be conducted, and (3) some of the traits are difficult and expensive to assess, such as microfibril angle and cell wall thickness.

Traditionally, the selection of trees with desirable attributes for breeding or propagation has been based on both pedigree and phenotypic information. The genetic merit of candidate trees in a breeding population is now estimated using individual tree model or the so-called animal model in a mixed model framework^5–7^. Hence, the performance of a candidate tree and all known pedigree relationships with other members of its population are used to estimate its breeding value (EBV). The model is characterized by the fitting of a random component for the breeding value of each individual, which can be obtained using Best Linear Unbiased Prediction (BLUP)^8^. The animal model can also account for other environmental and genetic effects^9^. The additive genetic relationships between individuals is of crucial importance in the prediction of breeding values. The probability of identical genes by descent occurring in any pair of individuals is called the coancestry^10^ and the additive genetic relationship is twice the coancestry. The genetic evaluation using BLUP is heavily dependent on the genetic covariance among individuals, both for accuracy and unbiased results^8^, and genetic covariance among individuals includes the additive genetic variance, the dominance variance and the epistatic variance. In the present article, we will focus on additive genetic variance only.

The additive genetic relationships among individuals are usually represented by a matrix called the Numerator Relationship Matrix or A-matrix. The inverse of this matrix is needed for solving the mixed model equations and for obtaining the best linear unbiased predictions of breeding values. Henderson^11^ proposed a recursive method to compute this matrix. The method is known as ABLUP. The A-matrix is symmetric. Its diagonal elements are equal to 1 + F_i_, where F_i_ is the inbreeding coefficient of the individual i, and its off-diagonal elements equal the numerator of the coefficient of relationship between individuals i and j^12^. The covariance among breeding values can be obtained by multiplying the additive genetic variance by the A-matrix. As the additive genetic relationship among pairs of relatives is estimated using registered pedigree information, all members of a family receive the same expected relationship (for instance, 0.25 for half-sibs, and 0.50 for full-sibs). Hence, it is neither possible to take account of Mendelian sampling, which is due to the random segregation of the alleles of the parents^13^ and may cause a deviation of actual relationship from the expected one, nor of potential pedigree identification errors or contamination (e.g.^14–17^). Moreover, Askew and El-Kassaby^18^ reported that for relatively undomesticated forest tree species, the average relationship does not allow detecting unknown population structure and/or inbreeding, as often shown for tree species (e.g.^14,19^). Thus, forest tree breeding values as well as heritability and genetic gain estimates obtained only with pedigree information could be biased.

In the early 2000’s, Meuwissen et al*.*^20^, in their seminal paper, proposed using genome-wide distributed markers to model the entire complement of QTL effects across the genome, whether these effects are significant or not, and to obtain genomic-estimated breeding values (GEBVs). This method is called genomic selection (GS). Since then, it has been tested for the selection of complex traits in numerous species, such as maize and wheat^21^, trees^22,23^, and cattle^24^. In the last two decades, there has been a rapid development in DNA marker technologies. The availability of large numbers of markers distributed genome-wide and relatively inexpensive high-throughput genotyping technologies offers the possibility to improve the efficiency of tree breeding at a reasonable cost^25–29^. Hence, depending on availability of markers for a given species, a large number of individuals can be genotyped for up to many thousands of markers. It is then possible to obtain genotypes from many different loci well distributed across all the chromosomes of a species (e.g.^17,30^).

Various statistical methods have been developed for GS and they can be classified in two main groups^31^. The methods of the first group are based on the concept that it is possible to predict the genetic value of individuals by using a regression model that relates phenotypes to all available markers. When the number of available markers generally exceeds the number of individuals in the population used to solve the regression, and the predictors (markers) are highly correlated, variable selection or shrinkage estimation procedures are required. Hoerl and Kennard^32^ proposed a method called ridge regression, which introduces a little bias so that the variance can be substantially reduced, which leads to a lower overall mean squared error. Tibshirani^33^ introduced the least absolute shrinkage and selection operator (LASSO) as an alternative to ridge regression. Since then, Bayesian estimation procedures of the shrinkage estimation methods have also been proposed to address the problem of multi-collinearity. With these regression methods, the genetic effect of each marker can be estimated and the summation of these marker effects for a given individual corresponds to its GEBV. The second group of methods uses the genomic relationships between individuals of a population (G-matrix), which are derived from their multi-locus genotypes, in a linear mixed or animal model framework to directly estimate the GEBV of any individual. It is usually referred to as Genomic Best Linear Unbiased Prediction or simply GBLUP. It is possible to use it in the context of an additive infinitesimal model where the standard pedigree-based numerator relation matrix (A-matrix) is replaced by the marker-based or realized genomic relationship matrix (G-matrix)^31,34^. Thus, this second marker-based method is more familiar to tree breeders and quantitative geneticists, and its results are easier to interpret for them.

More recently, a new method, which makes it possible to combine in a single GS analysis trees that were genotyped and trees for which only the pedigree information is known, has been proposed^35^. This method is referred as to the single-step GBLUP. A relationship matrix called H-matrix is generated using information from both the A-matrix and the G-matrix, and then again, as for GBLUP, it is possible to work in the context of an additive infinitesimal model, by simply replacing the A-matrix by the H-matrix.

Forest geneticists and tree breeders have used all these methods to analyze their data over the last decade. Depending of the genetic structure of the populations studied and the experimental design used, they reported estimates of heritability and genotype-by-environment (*G*x*E*) interaction for several quantitative traits based on additive effects only, and additive and non-additive effects (e.g.^2,28,36–39^). It is thus possible to make comparisons between results obtained with the pedigree-based and the marker-based approaches. Using a meta-analytical approach, our objective was thus to verify, for a large number of conifer and broadleaf tree species and traits, whether the estimates of narrow-sense heritability of quantitative traits as well as the expected genetic gains derived from selection were biased following an upward or downward trend when using registered pedigree information only, as compared to using marker-based information. We also wanted to compare the estimates of *G*x*E* interaction obtained with both approaches to determine if they provided the same results.

## Results

**Figure 1:**
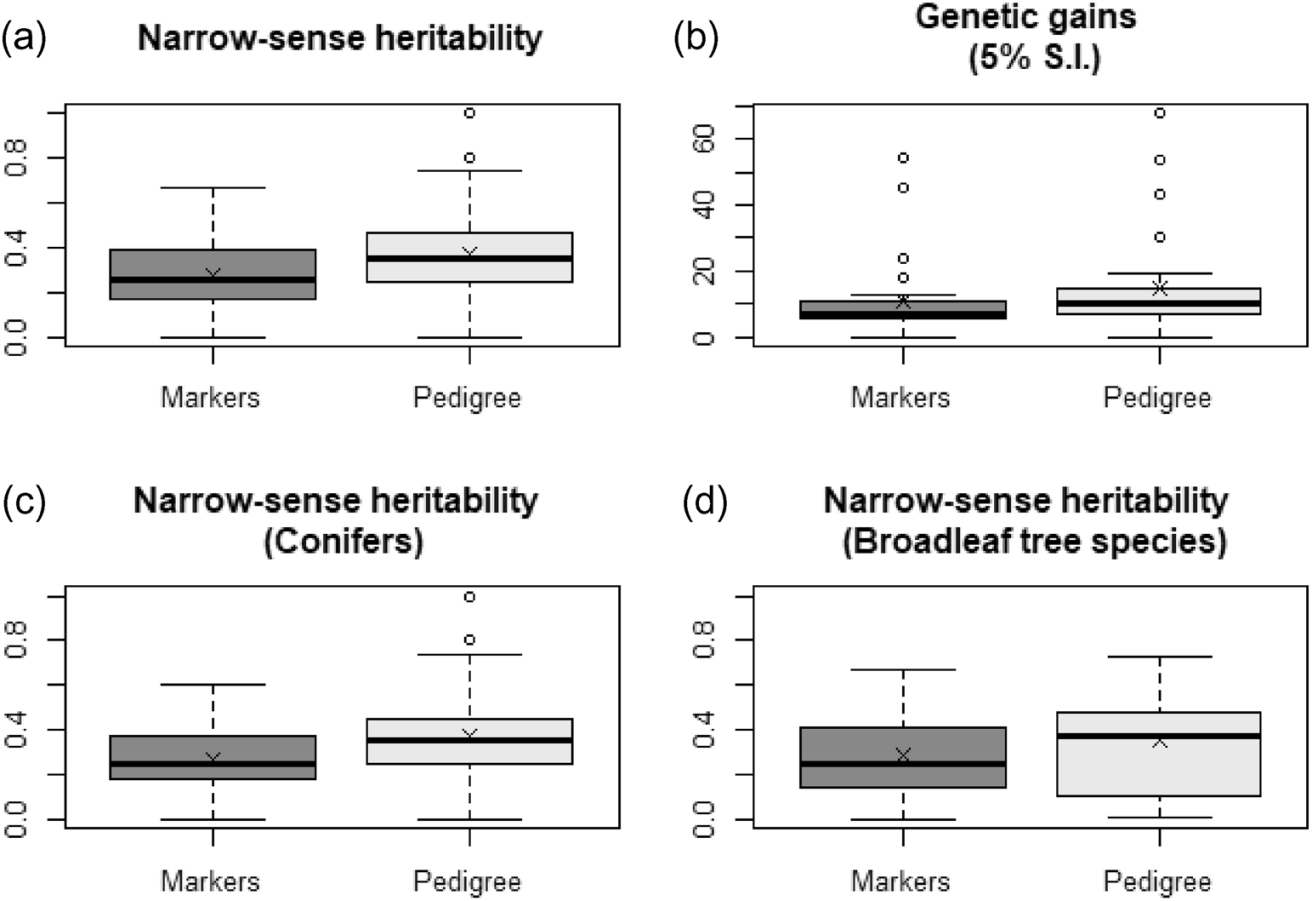
Pairwise comparisons between pedigree-based and marker-based estimates of (**a**) narrow-sense heritability (*h*^*2*^), (**b**) genetic gains in percentage (at 5% selection intensity, S.I.), and narrow-sense heritability (*h*^*2*^) for (**c**) conifers and (**d**) for broadleaf tree species. The symbol X represents the mean. The line in the box is the median. The box covers the first to the third quartiles. The dots are outliers, while the horizontal bars represent the minimum and the maximum values. Pedigree- and marker-based estimated genetic parameters and gains were significantly different based on the Wilcoxon matched-pairs signed-ranks tests.

Most of the published studies reported results for both growth and wood quality traits (Table 1 and Supplementary material 1). In a few of the studies, heritability estimates were also presented for insect resistance traits^1,2^. Narrow-sense heritability estimates were available for 87 study-trait pairs of pedigree-based and marker-based estimates. In total, this metadata sample represents estimates obtained from 16 distinct species or taxa, corresponding to 23 distinct tree breeding populations. Using a Wilcoxon signed-rank test, we found significant differences between the pedigree-based and the marker-based narrow-sense heritability estimates (p-value = 3.43e−09) when all the traits in all studies were considered, with pedigree-based estimates being higher than marker-based ones (Fig. 1a). The same trend was also observed for growth traits (p-value = 2.87e−05) and wood quality traits (p-value = 7.92e−05), when analyzed separately. When considering only conifers (57 study-trait pairs), the pedigree-based narrow-sense heritability estimates were again highly significantly higher than those obtained with DNA marker information (p-value = 4.23e−08; Fig. 1c). This trend was also observed for broadleaf species studies, although the difference was less significant (p-value = 0.0175; Fig. 1d). Genetic gains after selection of the 5% top trees for a variety of growth, wood quality and insect resistance traits were also provided in seven studies on spruces. A comparison of both the pedigree-based and the marker-based estimated genetic gains using a Wilcoxon signed-rank test again showed that globally, the gains estimated using the pedigree information only, were higher than those estimated using DNA marker information (p-value = 0.0021) (Fig. 1b).

**Table 1:** Pedigree-based and marker-based estimates of narrow-sense heritability for a variety of traits of conifer and broadleaved tree species.

Species	Material	Trait^a^	Heritability (*h*^*2*^)^b^		Genetic gain in percent (5% selection intensity)^b^		Reference
			Pedigree-based	Marker-based	Pedigree-based	Marker-based	
*Picea glauca*	Half-sibs	22-yr height	0.25	0.16	12.1	5.6	^23^
		22-yr wood density	0.39	0.24	7.9	2.3	
		22-yr wood stiffness	0.31	0.23	13.2	6.0	
		22-yr microfibril angle	0.38	0.24	14.4	5.4	
	Full-sibs	17-yr height	0.31 (0.09)	0.12 (0.04)	11.4	5.8	^28^
		17-yr DBH	0.15 (0.07)	0.09 (0.04)	9.3	5.5	
		7-yr wood density	0.25 (0.07)	0.33 (0.04)	7.1	6.4	
		17-yr microfibril angle	0.24 (0.07)	0.18 (0.04)	5.7	4.5	
	Full-sibs	16 to 28-yr height	0.26 (0.06)	0.25 (0.04)	7.6	7.2	^1^
		16 to 28-yr DBH	0.13 (0.04)	0.13 (0.04)	5.7	6.6	
		16 to 28-yr acoustic velocity	0.54 (0.12)	0.41 (0.08)	10.3	8.7	
		16 to 28-yr needle piceol	0.57 (0.12)	0.43 (0.08)	43.4	26.6	
		16 to 28-yr needle pungenol	0.70 (0.13)	0.47 (0.08)	53.4	45.4	
	Polycross	19-yr height	0.30 (0.11)	0.20 (0.06)	5.8 (0.04)	6.4 (0.10)	^17^
		19-yr DBH	0.27 (0.11)	0.21 (0.06)	6.8 (0.10)	9.3 (0.16)	
		18-yr wood density	0.42 (0.13)	0.37 (0.06)	4.4 (0.05)	5.9 (0.05)	
		19-yr wood stiffness	0.48 (0.14)	0.41 (0.06)	5.4 (0.05)	10.1 (0.12)	
*Picea mariana*	Full-sibs	25-yr height	0.68	0.42	13.1	13.0	^30^
		25-yr DBH	0.57	0.29	14.4	12.8	
		25-yr wood density	0.41	0.39	8.4	8.6	
		25-yr microfibril angle	0.74	0.43	14.9	12.8	
	Half-sibs	25-yr height	1.00 (0.20)	0.55 (0.09)	19.1	10.9	This study
		25-yr DBH	1.00 (0.00)	0.60 (0.08)	30.6	17.9	
		25-yr wood density	0.80 (0.21)	0.23 (0.10)	11.6	4.0	
		25-yr microfibril angle	0.44 (0.20)	0.14 (0.09)	18.2	6.7	
*Picea abies*	Full-sibs	15-yr height	0.47 (0.16)	0.22 (0.08)	16.0	8.0	^2^
		15-yr DBH	0.00 (0.00)	0.00 (0.00)	0.0	0.0	
		15-yr wood density	0.25 (0.11)	0.26 (0.08)	4.1	5.1	
		15-yr microfibril angle	0.08 (0.06)	0.06 (0.05)	7.7	9.1	
		16-yr acoustic velocity	0.37 (0.12)	0.29 (0.08)	7.3	7.1	
		Cumulative weevil attacks	0.47 (0.12)	0.27 (0.07)	68.0	54.6	
	Full-sibs	17-yr height	0.13 (0.04)	0.15 (0.05)			^45^
		30-yr wood density (pylodin)	0.41 (0.09)	0.34 (0.06)			
		30-yr acoustic velocity	0.45 (0.09)	0.37 (0.06)			
		30-yr wood stiffness	0.44 (0.09)	0.36 (0.06)			
*P. glauca* × *P. engelmannii*	Half-sibs	38-yr height	0.35 (0.14)	0.20 (0.06)			^43^
		38-yr DBH	0.05 (0.08)	0.07 (0.06)			
		38-yr wood density	0.38 (0.14)	0.18 (0.06)			
		38-yr wood stiffness	0.28 (0.12)	0.12 (0.06)			
*Pinus radiata*	Full-sibs	8-yr branch cluster	0.17	0.18			^59^
		8-yr stem straightness	0.13	0.09			
*Pinus sylvestris*	Full-sibs	10-yr height	0.19 (0.06)	0.16 (0.06)			^60^
		30-yr height	0.40 (0.09)	0.37 (0.08)			
		30-yr DBH	0.24 (0.07)	0.23 (0.07)			
		36-yr DBH	0.20 (0.07)	0.22 (0.07)			
		40-yr microfibril angle	0.28 (0.08)	0.24 (0.07)			
		40-yr wood stiffness	0.39 (0.10)	0.39 (0.09)			
		40-yr wood density	0.44 (0.10)	0.40 (0.09)			
		40-yr acoustic velocity	0.46 (0.10)	0.38 (0.08)			
*Pinus taeda*	Full-sib clones	6-yr height	0.32 (0.02)	0.31 (0.02)			^36,50^
		6-yr DBH	0.26 (0.07)	0.31 (0.02)			
*Pinus contorta*	Full-sibs & half-sibs	10-yr height	0.24 (0.07)	0.25 (0.06)			^61,62^
		12-yr wood density	0.57 (0.11)	0.47 (0.06)			
		12-yr microfibril angle	0.28 (0.08)	0.30 (0.07)			
*Pseudotsuga menziesii*	Full-sibs	12-yr height	0.27	0.17			^63^
		35-yr height	0.24	0.17			
		38-yr wood density	0.43	0.43			
*Eucalyptus nitens*	Half-sibs	6-yr height	0.09 (0.09)	0.08 (0.05)			^64^
		6-yr DBH	0.09 (0.09)	0.08 (0.05)			
		6-yr wood density	0.44 (0.13)	0.46 (0.07)			
		6-yr wood stiffness (1st log)	0.24 (0.11)	0.29 (0.07)			
*Ecalyptus benthamii*	Half-sibs	4.6-yr height	0.09	0.00			^65^
		4.6-yr DBH	0.33	0.18			
		4.6-yr volume	0.30	0.14			
*Eucalyptus pellita*	Half-sibs	54-month height	0.49 (0.19)	0.13 (0.06)			^66^
		54-month DBH	0.01 (0.08)	0.07 (0.05)			
		61-month kraft pulp yield	0.43 (0.18)	0.10 (0.06)			
*E. grandis* × *E. urophylla*	Full-sib hybrids	3-yr height	0.42	0.41			^44^
		3-yr wood density	0.59	0.56			
		3-yr height	0.48	0.39			
		3.7-yr wood density	0.42	0.34			
	Outbred F2 full-sib hybrids	3-yr mean annual increment	0.33	0.26			^67^
		3-yr basic wood density	0.69	0.67			
		5-yr screened pulp yield	0.46	0.37			
	Full-sib hybrids	6-yr height	0.10 (0.05)	0.19 (0.05)			^51^
		6-yr circumference at breast height	0.09 (0.04)	0.18 (0.04)			
		5-yr basic wood density	0.23 (0.04)	0.35 (0.05)			
		5-yr pulp yield	0.27 (0.05)	0.46 (0.05)			
*E. grandis* × *E. urophylla, E.grandis* × *camaldulensis*	F1 hybrids, Backcross, F2 hybrids	5-yr height	0.09 (0.13)	0.14 (0.06)			^68^
		5-yr DBH	0.41 (0.15)	0.23 (0.07)			
		5-yr mean annual increment	0.45 (0.14)	0.21 (0.07)			
		5-yr wood density	0.70 (0.16)	0.57 (0.05)			
		5-yr microfibril angle	0.11 (0.11)	0.13 (0.09)			
*E. urophylla* × *E. grandis*	Full-sib hybrid clones	32-month height	0.17 (0.07)	0.15 (0.07)			^69^
*Populus nigra* × *P. deltoides*	Full-sib hybrid clones	1-yr height	0.61	0.40			^70^
		2-yr height	0.73	0.51			
		2-yr stem circonference	0.73	0.51			

^a^DBH = diameter at breast height.

^b^When reported in the original papers, standard errors in parentheses.

**Figure 2:**
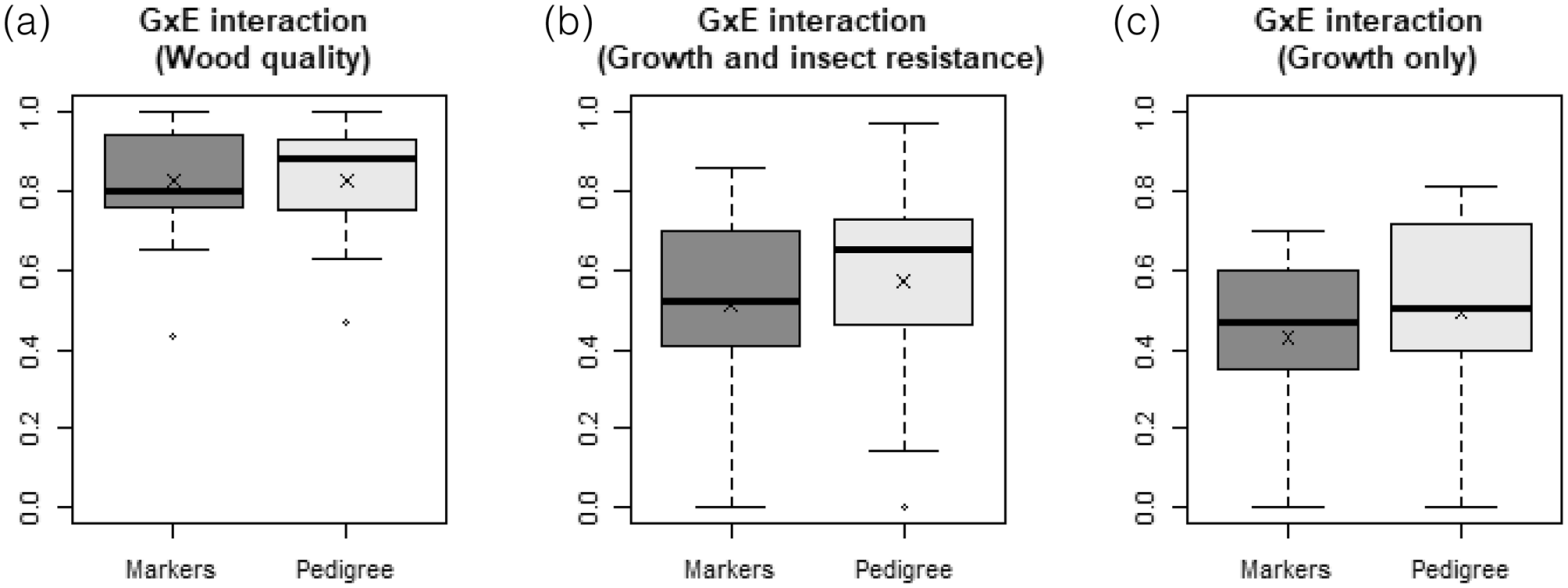
Genotype-by-environment (*G*x*E*) interaction for (**a**) wood quality, (**b**) growth and insect resistance, and (**c**) only growth traits in conifers. *G*x*E* interaction was not presented separately for insect resistance because the sample size was too small to obtain meaningful statistical tests. For *G*x*E*, the values of type-B genetic correlations are shown, which are inversely correlated to the amplitude of *G*x*E* interaction. The symbol X represents the mean. The line in the box is the median. The box covers the first to the third quartiles. The dots are outliers, while the horizontal bars represent the minimum and the maximum values. Pedigree- and marker-based estimated *G*x*E* interactions are significantly different based on the Wilcoxon matched-pairs signed-ranks tests, except for wood quality traits.

In a few studies and for several traits, results of analyses carried out on more than one site were reported. In such cases, estimates of *G*x*E* interaction using type-B genetic correlations were presented, which are inversely correlated to the amplitude of *G*x*E* interaction. We found such estimates in six of the studies reported in the literature (Table 2). Although we could observe variation in the estimates between pedigree-based and marker-based methods, we could not find an overall significant difference (p-value = 0.0644) using a Wilcoxon signed-rank test. However, this result clearly depended on the type of traits analyzed. Indeed, when the two approaches were compared for wood quality traits only, there was no significant difference between them (p-value = 0.9645) (Fig. 2a). On the other hand, statistical significance was reached for the other traits (growth and resistance to insects pooled together, p-value = 0.0107) (Fig. 2b), with lower type-B genetic correlations and thus higher *G*x*E* interaction estimates obtained with the information based on DNA markers. Only two studies considered insect resistance traits so no formal testing could be conducted for these traits only. When considering only growth traits, the statistical difference between pedigree-based and marker-based methods was of the same order of magnitude (p-value = 0.0206) (Fig. 2c) as that obtained by regrouping growth and insect resistance traits (Fig. 2b). This contrasting result between wood quality traits and the other traits may not be surprising, given that wood quality traits are generally under stronger genetic control than the other traits, and thus less influenced by the environment^40–42^. Therefore, the absence of significant difference between the two approaches when all traits were considered together was because wood quality traits, which represented 58% of the study-trait pairs analyzed, are clearly less influenced by *G*x*E* interaction.

**Table 2:** Pedigree-based and marker-based estimates of genotype-by-environment (*G*x*E*) interaction for a variety of traits of conifer tree species.

Species	Material	Population size parents/families/progenies	Trait^a^	*G*x*E*^b^		References
				Pedigree-based	Marker-based	
*Picea glauca*	Full-sibs	39/59/1748	17-yr height	0.73 (0.12)	0.60 (0.17)	^28^
			17-yr DBH	0,53 (0.19)	0.48 (0.20)	
			7-yr wood density	0.94 (0.08)	0.95 (0.06)	
			17-yr microfibril angle	1.00 (0.00)	1.00 (0.14)	
	Full-sibs	212/136/1516	16 to 28-yr height	0.40 (0.17)	0.35 (0.15)	^1^
			16 to 28-yr DBH	0.14 (0.28)	0.17 (0.24)	
			16 to 28-yr acoustic velocity	0.63 (0.16)	0.65 (0.16)	
			16 to 28-yr piceol	0.80 (0.11)	0.80 (0.13)	
			16 to 28-yr pungenol	0.72 (0.12)	0.70 (0.13)	
	Polycross	42/54/1513	19-yr height	0.72 (0.21)	0.60 (0.16)	^17^
		54/38/892	19-yr DBH	0.81 (0.25)	0.70 (0.17)	
			18-yr wood density	0.93 (0.19)	1.00 (0.00)	
			19-yr wood stiffness	0.93 (0.16)	0.92 (0.09)	
*Picea abies*	Full-sibs	35/40/726	15-yr height	0.65 (0.15)	0.52 (0.17)	^2^
			15-yr DBH	0.00 (0.00)	0.00 (0.00)	
			15-yr wood density	0.65 (0.20)	0.76 (0.17)	
			15-yr microfibril angle	0.47 (0.32)	0.43 (0.32)	
			16-yr acoustic velocity	0.79 (0.15)	0.76 (0.16)	
			Cumulative weevil attacks	0.97 (0.08)	0.86 (0.15)	
	Full-sibs	55/128/1370	17-yr height	0.48	0.41	^45^
			30-yr wood density (pylodin)	0.88	0.90	
			30-yr acoustic velocity	0.88	0.80	
			30-yr wood stiffness	0.88	0.94	
*Pinus contorta*	Full-sibs & half-sibs	57/42/1429	10-yr height	0.46 (0.24)	0.46 (0.20)	^61,62^
			12-yr wood density	0.87 (0.10)	0.78 (0.11)	
			12-yr microfibril angle	1.00 (-)	1.00 (-)	

^a^DBH = diameter at breast height.

^b^When reported in the original papers, standard errors in parentheses.

However, even for growth traits, the importance of *G*x*E* interaction appears to depend on the species and the populations considered. For instance, substantial *G*x*E* interactions were reported for interior spruce from western Canada^43^, for loblolly pine from southeastern United States^44^, and for Norway spruce in Sweden^45^. Moreover, these authors have indicated that GS models ideally needed to be calibrated for each breeding zone. In contrast to these results, much lower *G*x*E* interactions were reported for black spruce^30,46^, even for growth traits. Similarly, it was shown that *G*x*E* interaction was also limited in white spruce large genetic trials established in eastern Canada^28,47^. Nevertheless, the sole use of pedigree-based information appeared to underestimate the importance of *G*x*E* interaction in most studies especially for lower heritability traits, as compared to using marker-based methods (Table 2).

## Discussion

Results of the present meta-analytical study provides clear evidence that quantitative genetic analyses based on registered pedigree information only, resulted in upwardly biased estimates of narrow-sense heritability for growth, insect resistance, and wood quality traits in forest tree species, as compared to estimates obtained with realized genomic relationships based on DNA markers sampled genome-wide. For open-pollinated families, additive genetic variance estimates can be biased upwardly as the assumption of true half-siblings cannot be confirmed^48,49^. Several authors have indeed reported such bias^1,43^. However, this is the first time that a metadata analysis brings evidence of this bias even with full-sib family pedigree structure, although it has already been suspected in a few previous studies (e.g.^36,50^). Indeed, when we removed results of studies on open-pollinated families (22 study-trait pairs) in our metadata analysis, the Wilcoxon signed-rank test still remained highly significant (p-value = 5.64e−06), thus confirming the observed general bias of using registered pedigree information to represent the genetic relationships existing between the individuals making up the breeding population analyzed. The same upwardly biased trend was also observed for genetic gain estimates derived from pedigree information only, with important implications for conventional tree breeding programs.

The same significant trend was observed in both conifers and broadleaf species, although it was weaker in the latter. Several reasons may explain this difference. For conifers, 48 out of the 57 species- trait pairs compared (84%) showed higher pedigree-based heritability estimates than marker-based estimates. For broadleaf species, 22 of the 30 species-traits pairs (73%) showed such results. Thus, the proportion of significant higher pedigree-based estimates was marginally reduced in broadleaf species. The smaller number of species-trait pairs available for broadleaf species as compared to those of conifers may also partly explain the reduction in the significance of the statistical test. Indeed, the Wilcoxon matched-pairs signed-ranks test gives more weight to a pair that shows a large difference between the two conditions compared than a pair that shows a small difference. For many of the species-trait pairs of the broadleaf species, the differences between both the pedigree-based and the marker-based heritability estimates were quite small, but the trend remained statistically significant, as that observed for conifers. In addition, one can observe that the heritability estimates obtained with the pedigree information only were lower to those from marker information for all species-trait pairs in a single study on 6-yr old *E. grandis* x *E. urophylla* full-sibs^51^. After a thorough analysis, these authors concluded that putative pedigree errors (pollen contamination and mislabeling) negatively affected their ability to estimate accurate heritability estimates of traits based on pedigree information only. This might be the best explanation, knowing that in another study on the same hybrid species^44^, such lower pedigree-based heritability estimates were not observed. As this study provided half of the 8 species-trait pairs that did not follow the general trend of higher pedigree-based heritability estimates, it had some influence on the lower significance of the statistical test.

While pedigree records and performance data for dairy cattle date back to the late 1800s, together with widespread collection of performance data shortly thereafter^52^, forest tree breeding programs are generally only in their second or third generation at best^4^, and the available pedigree records and performance data are scarce. Thus, the presence of any preexisting inbreeding or relatedness among the ancestors of the current generation in typical forest genetic experiments is most often unknown unless marker-based assessment is used^18,19^. Consequently, using the current generation average (theoretical) additive genetic relationships between individuals does not allow taking account of ancestral effects. This may partly explain the systematic overestimation of the real additive genetic relationships observed in this study when using the pedigree-based approach to estimate breeding values and associated genetic parameters in forest trees.

The DNA markers used to estimate identity-by-state relatedness between individuals represent the observed (realized genomic relationships) rather than the average (theoretical) relationship values and thus, make it possible to potentially capture distant relationships and the variation around close relationships due to Mendelian sampling^34,35^. Hence, more accurate additive genetic variance and breeding values can be obtained. As indicated by several authors^28,53^, GS can have a substantial impact on the rate of genetic gains, especially because the use of realized genomic relationships is associated with increased accuracy in estimating the additive genetic variance and the breeding values.

One could wonder if the combination of both the pedigree and marker information would help obtain more accurate genetic parameter estimates. Single-step GBLUP analysis or the use of blended relationship matrices (H-matrix), which makes it possible to carry out an analysis combining both genotyped individuals and those for which only the registered pedigree is available, was proposed to take advantage of all the information available^35^. This approach would likely be useful when the number of genotyped individuals is limited. In such non-optimal conditions, the marker-based estimates would likely not be more accurate than those obtained with the registered pedigree information would, and combining both types of data might be somewhat advantageous, but we would not recommend it outright. When the marker density and genome coverage are inadequate or the number of genotyped individuals is small relative to the non-genotyped individuals, estimates obtained would likely be closer to the upwardly biased pedigree-based genetic parameters, because the information derived from genomic data would be insufficient to counterbalance the bias from using pedigree information. Thus, the best option would still be the use of genomic-based approaches applied to most or all of the population even in a situation where all pedigree errors could be recovered by pedigree reconstruction, given that genomic-based approaches also take into account the effects of Mendelian sampling.

One interesting finding of the current metadata analysis is that the pedigree-based approach appears to underestimate the importance of *G*x*E* interaction as compared with marker-based methods for traits that respond more strongly to variation in environmental conditions. To delineate breeding zones and select superior trees to assemble their breeding and production populations, tree breeders have traditionally based their decisions mainly on growth and adaptive traits. These traits are generally under low to moderate genetic control and thus, are more influenced by the environment. Hence, the use of a marker-based approach to estimate more precisely *G*x*E* interaction would be beneficial, especially to tree breeders who have to address the reforestation and plantation needs of land or territories of more heterogeneous nature. The accurate prediction of trait value and genetic merit to specific environments is becoming even more important for some tree breeders given the context of deploying efficient climate change mitigation measures such as seed transfer and assisted migration.

In addition to obtaining more precise estimates of genetic parameters and gains, GS offers other significant advantages over conventional breeding based on registered pedigree information. It indeed makes it possible to practice higher selection intensities or facilitate multi-trait selection by screening large number of candidates without phenotyping all of them or even phenotyping only a fraction of them^2^. It also allows considering forward selection of superior individuals at an earlier stage and thus to hasten breeding cycles^17,54,55^. This increased flexibility and efficiency will also be proving particularly important in the context of climate change, to allow tree breeders to adjust their selection goals more rapidly. Consequently, while being more accurate, the genetic gains per time unit provided by the use of GS are considerably increased as well as the benefits and profitability of tree breeding programs^56^. Although our study was specifically on forest tree species, we believe that our results might also have similar implications for breeders working with other plant species that are in their first steps of domestication.

Overall, tree breeders should take advantage of the reduction of breeding cycles and the increase in accuracy of genetic parameters and genetic gain estimates resulting from the use of GS approaches. Over the last decade, the cost of genotyping offered by commercial high-throughput genotyping platforms has also been regularly decreasing so that such an investment should now be viewed as affordable and essential for the rightful management and renewed progress of tree breeding programs.

## Material and methods

We conducted a meta-analytical review of scientific papers on tree genomic selection in which both marker-based and pedigree-based estimates of narrow-sense heritability were reported. We found 22 studies that were carried out over the last 10 years (Table 1 and Supplementary material 1). Among these, 14 were carried out on conifer breeding populations, whereas the eight remaining ones were mainly proof-of-concept studies for eucalypts. Results of several additional GS studies on forest tree species have also been reported. However, as pedigree-based genetic parameters were not presented along with marker-based estimates, it was not possible to include these additional studies in the current metadata analysis. These studies also relied on various marker-based methods and in most cases, whatever the method used, the results were similar. Thus, when results were reported for GBLUP as well as for other marker-based methods, we preferentially presented estimates obtained with the GBLUP method in Table 1. When GBLUP results were not available, we indicated the marker-based method used. We also listed in Supplementary material 1 all marker-based methods tested in the various studies. GBLUP was also the marker-based method that we used for a new study that we conducted on an open-pollinated family test of black spruce (*Picea mariana* (Mill.) B.S.P.) that we incorporated in the present analysis (see Table 1, and Supplementary material 2). To determine whether the genetic parameters estimated with the ABLUP method were different from those obtained with a marker-based method, were carried out non-parametric Wilcoxon matched-pairs signed-ranks tests using the wilcox.test function in the R v.3.6.1 environment^57^.

We opted for a non-parametric test procedure because the genetic parameters compared were obtained using different genetic material as well as experimental designs and marker types. Use of a parametric test would have required that data meet some assumptions, such as that differences in the matched-pairs follow a normal distribution and that the sample of pairs is a random sample for its population. In the context of the present metadata analysis, these assumptions could not be met adequately. The Wilcoxon matched-pairs signed-ranks test^58^ is a non-parametric test procedure that gives more weight to a pair that shows a large difference between the two conditions compared than a pair that shows a small difference. This test makes it possible to tell which member of a pair is greater and to rank the differences in order of absolute size. With such a test, we could identify for each pair which member is greater, and we could make that judgment globally for the entire sample of matched pairs as well.

## Supplementary Information

Supplementary Information.
