# Supplementary material for: Metadata analysis indicates biased estimation of genetic parameters and gains using conventional pedigree information instead of genomic-based approaches in tree breeding
Source: Sci Rep. 2022 Mar 10;12:3933. doi: 10.1038/s41598-022-06681-y (PMC8913692; doi:10.1038/s41598-022-06681-y)
Supplement: Supplementary file 1 — Supplementary Information. [file 41598_2022_6681_MOESM1_ESM.pdf]

Supplementary material 1.

Table 1. Pedigree-based and marker-based estimates of narrow-sense heritability for a variety of traits of conifer and broadleaved tree species, including information on the population make-up, and the type of model and the number of markers used.

Species						Heritability (h^2)[†]		Genetic gain in percent (5% selection intensity)[†]		Reference
	Material	Population size parents/families/progenies	Marker-based [‡]	Number of markers	Trait[a]	Pedigree-based	Marker-based	Pedigree-based	Marker-based	
Picea glauca										
	Half-sibs	unknown/214/1694	Bayesian RR[‡] BLASSO	6385	22-yr height	0.25	0.16	12.1	5.6	23
					22-yr wood density	0.39	0.24	7.9	2.3	
					22-yr wood stiffness	0.31	0.23	13.2	6.0	
					22-yr microfibril angle	0.38	0.24	14.4	5.4	
	Full-sibs	39/59/1748	Bayesian RR BayesCπ[‡]	6932	17-yr height	0.31 (0.09)	0.12 (0.04)	11.4	5.8	28
					17-yr DBH	0.15 (0.07)	0.09 (0.04)	9.3	5.5	
					7-yr wood density	0.25 (0.07)	0.33 (0.04)	7.1	6.4	
					17-yr microfibril angle	0.24 (0.07)	0.18 (0.04)	5.7	4.5	
	Full-sibs	212/136/1516	GBLUP	4148	16 to 28-yr height	0.26 (0.06)	0.25 (0.04)	7.6	7.2	1
					16 to 28-yr DBH	0.13 (0.04)	0.13 (0.04)	5.7	6.6	
					16 to 28-yr acoustic velocity	0.54 (0.12)	0.41 (0.08)	10.3	8.7	
					16 to 28-yr needle piceol	0.57 (0.12)	0.43 (0.08)	43.4	26.6	
					16 to 28-yr needle pungenol	0.70 (0.13)	0.47 (0.08)	53.4	45.4	
	Polycross	42/54/1513 54/38/892	GBLUP	4092	19-yr height	0.30 (0.11)	0.20 (0.06)	5.8 (0.04)	6.4 (0.10)	17
					19-yr DBH	0.27 (0.11)	0.21 (0.06)	6.8 (0.10)	9.3 (0.16)	
					18-yr wood density	0.42 (0.13)	0.37 (0.06)	4.4 (0.05)	5.9 (0.05)	
					19-yr wood stiffness	0.48 (0.14)	0.41 (0.06)	5.4 (0.05)	10.1 (0.12)	
Picea mariana										
	Full-sibs	27/34/734	Bayesian RR	4993	25-yr height	0.68	0.42	13.1	13.0	30
					25-yr DBH	0.57	0.29	14.4	12.8	
					25-yr wood density	0.41	0.39	8.4	8.6	
					25-yr microfibril angle	0.74	0.43	14.9	12.8	
	Half-sibs	unknown/101/398	GBLUP	1114	25-yr height	1.00 (0.20)	0.55 (0.09)	19.1	10.9	This study
					25-yr DBH	1.00 (0.00)	0.60 (0.08)	30.6	17.9	
					25-yr wood density	0.80 (0.21)	0.23 (0.10)	11.6	4.0	
					25-yr microfibril angle	0.44 (0.20)	0.14 (0.09)	18.2	6.7	

Picea abies										
	Full-sibs	35/40/726	GBLUP[‡]	3914	15-yr height	0.47 (0.16)	0.22 (0.08)	16.0	8.0	2
			Threshold GBLUP		15-yr DBH	0.00 (0.00)	0.00 (0.00)	0	0	
			Bayesian RR		15-yr wood density	0.25 (0.11)	0.26 (0.08)	4.1	5.1	
			BayesCπ		15-yr microfibril angle	0.08 (0.06)	0.06 (0.05)	7.7	9.1	
					16-yr acoustic velocity	0.37 (0.12)	0.29 (0.08)	7.3	7.1	
					Cumulative weevil attacks	0.47 (0.12)	0.27 (0.07)	68.0	54.6	
	Full-sibs	55/128/1370	Bayesian RR	116765	17-yr height	0.13 (0.04)	0.15 (0.05)			45
			Bayesian LASSO		30-yr wood density (pylodin)	0.41 (0.09)	0.34 (0.06)			
			RKHS		30-yr acoustic velocity	0.45 (0.09)	0.37 (0.06)			
			GBLUP[‡]		30-yr wood stiffness	0.44 (0.09)	0.36 (0.06)			
P. glauca × *P. engelmannii*										
	Half-sibs	Unknown/25/1126	RR-BLUP	16383	38-yr height	0.35 (0.14)	0.20 (0.06)			43
			GRR		38-yr DBH	0.05 (0.08)	0.07 (0.06)			
			GBLUP[‡]		38-yr wood density	0.38 (0.14)	0.18 (0.06)			
					38-yr wood stiffness	0.28 (0.12)	0.12 (0.06)			
Pinus radiata										
	Full-sibs	53/52/465	GBLUP	67168	8-yr branch cluster	0.17	0.18			59
		24/54/523			8-yr stem straightness	0.13	0.09			
Pinus sylvestris										
	Full-sibs	40/183/694	Bayesian RR	8719	10-yr height	0.19 (0.06)	0.16 (0.06)			60
			Bayesian LASSO		30-yr height	0.40 (0.09)	0.37 (0.08)			
			GBLUP[‡]		30-yr DBH	0.24 (0.07)	0.23 (0.07)			
					36-yr DBH	0.20 (0.07)	0.22 (0.07)			
					40-yr microfibril angle	0.28 (0.08)	0.24 (0.07)			
					40-yr wood stiffness	0.39 (0.10)	0.39 (0.09)			
					40-yr wood density	0.44 (0.10)	0.40 (0.09)			
					40-yr acoustic velocity	0.46 (0.10)	0.38 (0.08)			
Pinus taeda										
	Full-sib clones	32/61/951	RR-BLUP	4853	6-yr height	0.32 (0.02)	0.31 (0.02)			36, 50
			GBLUP[‡]		6-yr DBH	0.26 (0.07)	0.31 (0.02)			
Pinus contorta										61, 62
	Full-sibs & half-sibs	57/42/1429	GBLUP[‡]	51213	10-yr height	0.24 (0.07)	0.25 (0.06)			
			BayesB		12-yr wood density	0.57 (0.11)	0.47 (0.06)			
			BayesC		12-yr microfibril angle	0.28 (0.08)	0.30 (0.07)			
Pseudotsuga menziesii										63

	Full-sibs	21*/37/1372	RR-BLUP‡	69551	12-yr height	0.27	0.17	
			GRR		35-yr height	0.24	0.17	
					38-yr wood density	0.43	0.43	
Eucalyptus nitens								
	Half-sibs	133*/47/431	GBLUP	12236	6-yr height	0.09 (0.09)	0.08 (0.05)	64
		65*/25/236			6-yr DBH	0.09 (0.09)	0.08 (0.05)	
					6-yr wood density	0.44 (0.13)	0.46 (0.07)	
					6-yr wood stiffness (1st log)	0.24 (0.11)	0.29 (0.07)	
Ecalyptus benthamii								
	Half-sibs	50*/30/505	GBLUP‡	13787	4.6-yr height	0.09	0.00	65
			Bayesian LASSO		4.6-yr DBH	0.33	0.18	
			BRR		4.6-yr volume	0.30	0.14	
			BayesA					
			BayesB					
			BayesCπ					
Eucalyptus pellita								
	Half-sibs	Unknown/28/423	GBLUP	2023	54-month height	0.49 (0.19)	0.13 (0.06)	66
					54-month DBH	0.01 (0.08)	0.07 (0.05)	
					61-month kraft pulp yield	0.43 (0.18)	0.10 (0.06)	
E. grandis X *E. urophylla*								
	Full-sib hybrids	11/43/738	RR-BLUP	1455	3-yr height	0.42	0.41	
		51/75/920			3-yr wood density	0.59	0.56	44
					3-yr height	0.48	0.39	
					3.7-yr wood density	0.42	0.34	
	Outbred F2 full-sib hybrids	10/37/768	GBLUP	24806	3-yr mean annual increment	0.33	0.26	67
					3-yr basic wood density	0.69	0.67	
					5-yr screened pulp yield	0.46	0.37	
	Full-sib hybrids		GBLUP‡	41304	6-yr height	0.10 (0.05)	0.19 (0.05)	51
			RR-BLUP		6-yr circumference at breast height	0.09 (0.04)	0.18 (0.04)	
			Bayesian LASSO		5-yr basic wood density	0.23 (0.04)	0.35 (0.05)	
			RKHS		5-yr pulp yield	0.27 (0.05)	0.46 (0.05)	

E. grandis X *E. urophylla, E.grandis* X E. camaldulensis

	F1 hybrids, Backcross, F2 hybrids	46/45/1000	GBLUP	33398	5-yr height	0.09 (0.13)	0.14 (0.06)	68
					5-yr DBH	0.41 (0.15)	0.23 (0.07)	
					5-yr mean annual increment	0.45 (0.14)	0.21 (0.07)	
					5-yr wood density	0.70 (0.16)	0.57 (0.05)	
					5-yr microfibril angle	0.11 (0.11)	0.13 (0.09)	
Ecalyptus urophylla X E. grandis								
	Full-sib hybrid clones	22/69/1130	GBLUP	3303	32-month height	0.17 (0.07)	0.15 (0.07)	69
Populus nigra x *P. deltoides*								
	Full-sib hybrid clones	24/35/1011	GBLUP‡	50565	1-yr height	0.61	0.40	70
			weighted GBLUP		2-yr height	0.73	0.51	
					2-yr stem circonference	0.73	0.51	

* effective population size

† when reported in the original papers, standard error in parentheses

‡ methods for which estimates were provide in the present study

[a] DBH = diameter at breast height

Supplementary material 2.

Design and methods for the original results we are reporting from a black spruce open-pollinated family test in the province of New Brunswick, Canada

Genetic trial and phenotyping

The black spruce open-pollinated progeny test was established in 1979 near Miramichi, New Brunswick, Canada. The test consisted of 130 open-pollinated families, laid out into 10 complete blocks with families planted in four-tree row-plots. Three blocks were selected for sub-sampling with very little heterogeneity for tree survival and previously measured tree size and diameter traits. In 2006, 398 individual trees (age 27) were selected from these blocks for collection of needles and increment cores at breast height. The objective was to have 101 families represented each by four trees.

Wood quality traits were obtained by scanning wood flitch samples prepared from collected increment cores using Silviscan (Evans and Downes, 2001) at FPInnovations (Vancouver, Canada). Pith to bark profiles were taken for density in 25µm steps using X-ray densitometry. Microfibril angles was measured in 1 mm steps using X-ray diffractometry (more details on SilviScan sample preparation and data acquisition can be found in Lenz et al. 2010). Tree height and diameter at breast height (DBH) were also measured in the field.

Genotyping

DNA samples for genotyping were isolated from needles by using the Qiagen DNeasy Plant Kit (Mississauga, ON, Canada) and quantified using the Pico- Green fluorescent dye (Thermo Fisher Scientific, Waltham, MA). Genotyping was conducted with an Illumina GoldenGate array (Shen et al., 2005) originally manufactured for 1536 SNPs representative of 633 candidate genes for wood formation, at the Genome Quebec Innovation Centre (McGill University, Montreal, Canada). Normalized intensity data was used to cluster and assign genotypes with the GenomeStudio^{TM} software (Illumina Inc., San Diego, CA), where SNPs with minimum *GenTrain* scores of 0.25 were considered valid together with a minimum call rate of 85%. The final dataset retained for further analyses consisted of 398 trees genotyped for 1114 SNPs, with average call rate over 99%.

Statistical analyses

For each trait, variance components, heritability, and breeding values were estimated using the conventional pedigree-based (ABLUP) and the genomic-based (GBLUP) individual-tree mixed models in ASReml-R v.4.1 (Butler et al. 2017):

$$y = \mu + Z_1 b + Z_2 \alpha + e,$$

where y is the phenotype; μ represents the overall mean; b is the random block effect, with $b \sim N(0, \sigma_b^2 I_b)$; α is the random additive genetic effect, with $\alpha \sim N(0, \sigma_a^2 A)$; and e is the residual term, with $e \sim N(0, \sigma_e^2 I_e)$. For the ABLUP method, the matrix A is the pedigree-based relationship matrix, which was replaced by the realized genomic relationship matrix G for the GBLUP method $\alpha \sim N(0, \sigma_a^2 G)$. The matrices Z_1 and Z_2 are incidence matrices of their corresponding effects.

Narrow-sense heritability estimates were obtained using the following formula:

$$\widehat{h^2} = \frac{\widehat{\sigma_a^2}}{\widehat{\sigma_a^2} + \widehat{\sigma_e^2}}$$

Standard errors of heritability estimates were obtained using the delta method.

References

Butler, D., Cullis, B. R., Gilmour, A. & Gogel, B. ASReml-R Reference Manual Version 4. (Hemel Hempstead, HP1 1ES, UK, 2017).

Lenz, P. R. N., Cloutier, A., MacKay, J. & Beaulieu, J. Genetic control of wood properties in Picea glauca – an analysis of trends with cambial age. *Can. J. For. Res.* **40**, 703-715 (2010).

Shen, R. *et al.* High-throughput SNP genotyping on universal bead arrays. *Mutation Research/Fundamental and Molecular Mechanisms of Mutagenesis* **573**, 70-82 (2005).
